# The cow udder as an influenza mixing ground

**DOI:** 10.7554/eLife.113101

**Published:** 2026-10-15

**Authors:** Silke Rimaux, Robert P de Vries

**Affiliations:** 1 Department of Chemical Biology and Drug Discovery, Utrecht Institute for Pharmaceutical Sciences, Utrecht University Utrecht Netherlands

**Keywords:** cow flu, pandemic, cow udder, bird flu, influenza A, strains, Viruses

## Abstract

Bovine mammary tissue can support infection and replication of diverse influenza A viruses and may provide an environment in which different viral strains can co-infect cells.

**Related research article** Pinto RM, Sharp CP, Beeson M, Pankaew N, Hassard JA, Moxom A, Magill C, Ross RA, Tuck L, Meek S, Lee HM, Jensen K, Dry I, Melo P, Yang J, Tan WS, Raut AA, Mishra A, de Wit S, Fitzgerald JR, Hope JC, Joanne Stevens S, Tom Burdon T, Sutton K, Esteves CL, Donadeu FX, Brown I, Barclay W, Peacock TP, Goldhill DH, Iqbal M, Murcia PR, Haslam SM, Gaunt E, Digard P. 2026. The cow udder is a potential coinfection site for influenza A viruses. *eLife* **15**:RP111076. doi: 10.7554/eLife.111076.

Avian flu is caused by the influenza A virus and mostly affects birds. But depending on its subtype, the virus can infect a wide range of host species, including mammals. Pigs are considered potential “gene mixing vessels” for multiple viral subtypes, as they can be infected with avian, swine, and human influenza A viruses.

Since 2020, the subtype H5N1 (clade 2.3.4.4b) has spread globally, causing mass mortalities in wild birds and poultry, and frequent transmission to several mammalian species, including occasional human infections (Peacock et al., 2025). In spring 2024, H5N1 was detected in dairy cattle in the United States, where the virus subsequently spread among herds across several states. This sustained circulation has raised questions about whether cattle may also provide opportunities for further viral adaptation and reassortment (Nguyen et al., 2025).

To date, two genotypes have been identified in US dairy cattle: B3.13, which was the first to infect dairy cows, and D1.1, which crossed the species barrier more recently in early 2025 (Mezhenskaia et al., 2026). Intriguingly, cows do not show respiratory symptoms. Instead, they develop an abrupt drop in milk production, which correlates with high levels of virus in the milk (Halwe et al., 2025). Human infections with ‘cow flu’ have also been reported, and laboratory studies have shown that bovine-derived isolates can replicate and cause disease in different animal models (Gu et al., 2024). However, human-to-human transmission has not yet been reported.

Now, in eLife, Rute Maria Pinto, Colin P. Sharp, Eleanor Gaunt, Paul Digard and colleagues report new insights on how the cow udder could serve as a mixing vessel for different strains of influenza A viruses (Pinto et al., 2026). The researchers, based at various research institutes in the United Kingdom, India and the Netherlands, infected different cow-derived cells with bovine, as well as avian, human and swine virus strains, and analyzed the virus’ ability to replicate and infect various bovine cells (Figure 1). The results showed that mammary cells could easily be infected by many different influenza A viruses. They then compared the fitness of bovine viruses with that of an avian precursor strain. Using several assays to measure viral replication, they found that three of the eight RNA gene segments of an influenza A virus (PB2, PA, and NS) improved the B3.13 virus’s ability to adapt to bovine cells (Dholakia et al., 2026).

**Figure 1.**
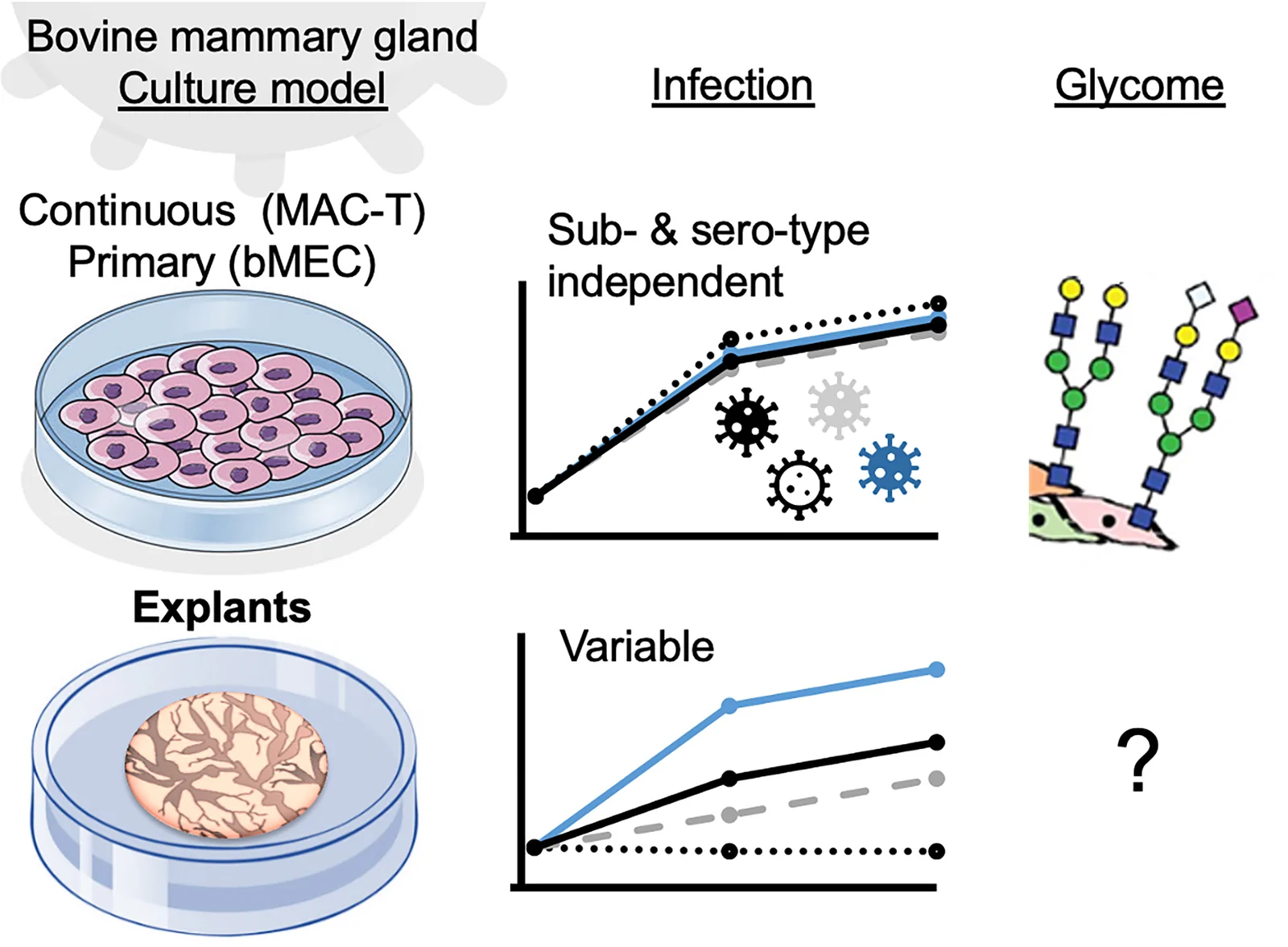
Properties of different bovine mammary gland culture models. Different approaches have been used to model the bovine mammary gland. These include two cell line models: a continuous cell line (MAC-T), which can be grown and divided repeatedly, and a primary bovine mammary cell line (bMEC) isolated from tissue. Infection of both cell lines supports efficient replication of influenza A viruses across different subtypes and serotypes. Mass spectrometry analysis revealed a complex glycome in both cell lines (top of the figure). When a tissue explant was infected with 2.3.4.4b viruses, viral replication efficiency differed between strains (bottom of the figure). The explant glycome has yet to be characterized.

Furthermore, Pinto et al. used reassortant or ‘mixed’ viruses containing two gene segments from one virus and six from another, carrying PR8 glycoproteins HA and NA as their surface proteins (2:6) – molecules that help the virus attach, enter, and leave host cells. This approach revealed that unadapted 2:6 2.3.4.4b strains, including avian Eurasian strains, could also replicate to high levels in cow mammary cells. These findings are consistent with other studies reporting the susceptibility of various bovine cell culture systems to different influenza viruses, including replication in bovine skin fibroblasts and mammary gland organoids (e.g., Turnbull et al., 2025; Fritsch et al., 2026).

Working with H5N1-infected, lactating cattle in high containment presents obvious infrastructural and logistical challenges. Thus, developing alternative bovine models that maintain biological relevance while reducing operational complexity is warranted. However, many models may lose some bovine characteristics in culture. When Pinto et al. infected mammary explants – the closest culture system to the actual udder – they found marked differences in viral quantities between bovine and precursor H5N1 strains. Thus, a bovine-adapted phenotype is relevant for achieving high viral loads in the cow udder. Noticeably, the viruses used in the infection experiments were 2:6 viruses again. The circulating bovine viruses have unique receptor-binding properties and are more promiscuous than classical H5 viruses before the introduction of the 2.3.4.4 antigenic clade (Good et al., 2024; Weber et al., 2026). It is, thus, unclear whether the different authentic H5N1 viruses would similarly infect these relevant cultures.

To further investigate the cow udder’s potential as a mixing vessel, the presence of both avian-type and human-type sialic acid receptors, which can serve as attachment points for influenza viruses, was examined using mass spectrometry. They found both receptor types, concluding that their infection experiments provide evidence that bovine cells can support infection with many different influenza A viruses. However, it would be useful to analyze the complete collection of sugars present on the cells and tissues of the explants and determine whether differences exist between cow tissues. Importantly, current mass spectrometry analyses have a clear caveat: they only represent possible glycan structures inferred from their measured masses rather than definitive structural assignments. Finally, a co-infection experiment in bovine cells using a human and an avian influenza strain produced a cell population infected with both viruses, providing an important proof of principle for co-infection and reassortment.

Taken together, the study by Pinto et al. supports the hypothesis that co-infections of human and avian viruses within the cow mammary glands are possible and thus reassortment events in the cow udder cannot be ruled out.

## References

[bib1] Dholakia V, Quantrill JL, Richardson SAS, Pankaew N, Brown MD, Yang J, Capelastegui F, Masonou T, Case KM, Ajeian J, Woodall MNJ, Magill C, Freimanis G, McCarron A, Staller E, Sheppard CM, Brown IH, Murcia PR, Smith CM, Iqbal M, Digard P, Barclay WS, Pinto RM, Peacock TP, Goldhill DH (2026). Polymerase mutations underlie early adaptation of H5N1 influenza virus to dairy cattle and other mammals. Nature Communications.

[bib2] Fritsch H, Arora P, Yan M, Grotha I, Pfarrer C, Becher P (2026). Beyond H5N1: influenza A virus infection in bovine udder organoids. Emerging Microbes & Infections.

[bib3] Good MR, Fernández-Quintero ML, Ji W, Rodriguez AJ, Han J, Ward AB, Guthmiller JJ (2024). A single mutation in dairy cow-associated H5N1 viruses increases receptor binding breadth. Nature Communications.

[bib4] Gu C, Maemura T, Guan L, Eisfeld AJ, Biswas A, Kiso M, Uraki R, Ito M, Trifkovic S, Wang T, Babujee L, Presler R, Dahn R, Suzuki Y, Halfmann PJ, Yamayoshi S, Neumann G, Kawaoka Y (2024). A human isolate of bovine H5N1 is transmissible and lethal in animal models. Nature.

[bib5] Halwe NJ, Cool K, Breithaupt A, Schön J, Trujillo JD, Nooruzzaman M, Kwon T, Ahrens AK, Britzke T, McDowell CD, Piesche R, Singh G, Pinho Dos Reis V, Kafle S, Pohlmann A, Gaudreault NN, Corleis B, Ferreyra FM, Carossino M, Balasuriya UBR, Hensley L, Morozov I, Covaleda LM, Diel DG, Ulrich L, Hoffmann D, Beer M, Richt JA (2025). H5N1 clade 2.3.4.4b dynamics in experimentally infected calves and cows. Nature.

[bib6] Mezhenskaia D, Babujee L, Lim A, Guan L, Nguyen D, Gu C, Neumann G, Poulsen K, Eisfeld AJ, Kawaoka Y (2026). Isolation and characterization of a clade 2.3.4.4b genotype D1.1 H5N1 virus from dairy cattle in Wisconsin. Journal of Virology.

[bib7] Nguyen TQ, Hutter CR, Markin A, Thomas M, Lantz K, Killian ML, Janzen GM, Vijendran S, Wagle S, Inderski B, Magstadt DR, Li G, Diel DG, Frye EA, Dimitrov KM, Swinford AK, Thompson AC, Snekvik KR, Suarez DL, Lakin SM, Schwabenlander S, Ahola SC, Johnson KR, Baker AL, Robbe-Austerman S, Torchetti MK, Anderson TK (2025). Emergence and interstate spread of highly pathogenic avian influenza A(H5N1) in dairy cattle in the United States. Science.

[bib8] Peacock TP, Moncla L, Dudas G, VanInsberghe D, Sukhova K, Lloyd-Smith JO, Worobey M, Lowen AC, Nelson MI (2025). The global H5N1 influenza panzootic in mammals. Nature.

[bib9] Pinto RM, Sharp CP, Beeson M, Pankaew N, Hassard JA, Moxom A, Magill C, Ross RA, Tuck L, Meek S, Lee HM, Jensen K, Dry I, Melo P, Yang J, Tan WS, Raut AA, Mishra A, de Wit S, Fitzgerald JR, Hope JC, Joanne Stevens S, Tom Burdon T, Sutton K, Esteves CL, Donadeu FX, Brown I, Barclay W, Peacock TP, Goldhill DH, Iqbal M, Murcia PR, Haslam SM, Gaunt E, Digard P (2026). The cow udder is a potential coinfection site for influenza A viruses. eLife.

[bib10] Turnbull ML, Zakaria MK, Upfold NS, Bakshi S, Magill C, Das UR, Clarke AT, Mojsiejczuk L, Herder V, Dee K, Liu N, Folwarczna M, Ilia G, Furnon W, Schultz V, Chen H, Devlin R, McCowan J, Young AL, Po WW, Smollett K, Yaseen MA, Ross R, Bhide A, van Kekem B, Fouchier RAM, da Silva Filipe A, Iqbal M, Roberts E, Hughes J, Werling D, Murcia PR, Palmarini M (2025). The potential of H5N1 viruses to adapt to bovine cells varies throughout evolution. Nature Communications.

[bib11] Weber J, Ponse NLD, Zhu X, Ríos Carrasco M, Han AX, Funk M, Lin T-H, Gabarroca García A, Spruit CM, Zhang D, Yu W, Wilson IA, Richard M, Boons G-J, de Vries RP (2026). The receptor binding properties of H5Ny influenza A viruses have evolved to bind to avian-type mucin-like O-glycans. PLOS Pathogens.

